# Spontaneously resolved invasive conjunctival squamous cell carcinoma with anterior chamber invasion in low-grade lymphoma patient^☆^

**DOI:** 10.1016/j.ajoc.2025.102348

**Published:** 2025-05-03

**Authors:** Tanya Boghosian, Promporn Patarajierapun, Ben J. Glasgow, Carla B. Berkowitz, Clemence Bonnet, Tara A. McCannel, Brad D. Barrows, Simon S.M. Fung

**Affiliations:** a Stein Eye Institute, University of California Los Angeles, Los Angeles, CA, 90095, United States; b Washington University in St. Louis School of Medicine, St. Louis, Missouri, 63110, United States; c Chulabhorn International College of Medicine, Thammasat university, Pathum Thani, Thailand; d Community Memorial Health System, Ventura, CA, 93003, United States; e Department of Ophthalmology, University of California San Francisco, San Francisco, CA, 94158, United States

**Keywords:** Ocular surface squamous neoplasia, Invasive conjunctival squamous cell carcinoma, Spontaneous regression

## Abstract

**Purpose:**

To report a case of invasive conjunctival squamous cell carcinoma (SCC) with anterior chamber invasion that resolved spontaneously after incisional biopsy and anterior chamber paracentesis in the absence of any treatment.

**Observations:**

A 76-year-old man presented with a 3-month history of a recurrent mass in the right eye. Examination showed an elevated, smooth amorphous lesion at the nasal conjunctivae, adherent to the scleral surface. There was also a pseudohypopyon at the angle adjacent to the lesion. Incisional biopsy of the right conjunctival lesion and intracameral fine-needle aspiration (FNA) were performed, which confirmed recurrent invasive SCC. Brachytherapy was planned but was deferred when spontaneous regression of the lesion was noted at the interim visit. The lesion completely resolved within one year post-biopsy with no additional interventions and has remained with no signs of recurrence two years after regression, permitting uneventful cataract surgery for visual rehabilitation.

**Conclusions and Importance:**

Invasive conjunctival squamous cell carcinoma is an advanced form of ocular surface neoplasia, typically necessitating extensive surgical excision and chemical or biologic therapy. This case highlights a potential pathway for the spontaneous resolution of squamous cell carcinoma post-incisional biopsy, although the precise underlying mechanisms remain unclear. Understanding these mechanisms could inform future treatment for squamous cell carcinoma and other neoplasms.

## 1 Introduction

Ocular surface squamous neoplasia (OSSN) encompasses a spectrum of neoplastic changes to the squamous epithelium of the cornea and conjunctiva. ^1^ Conjunctival squamous neoplasia, a subtype of OSSN, is graded based on histopathological examination depending on dysplastic involvement of the epithelium. ^2^^,^^3^ Here we describe a case of a patient with a history of chronic lymphocytic leukemia (CLL) and marginal zone lymphoma (MZL), who then developed recurrent biopsy-proven invasive conjunctival squamous cell carcinoma, which resolved spontaneously without further treatment 1 year after diagnosis.

## 2 Case report

**Fig. 1:**
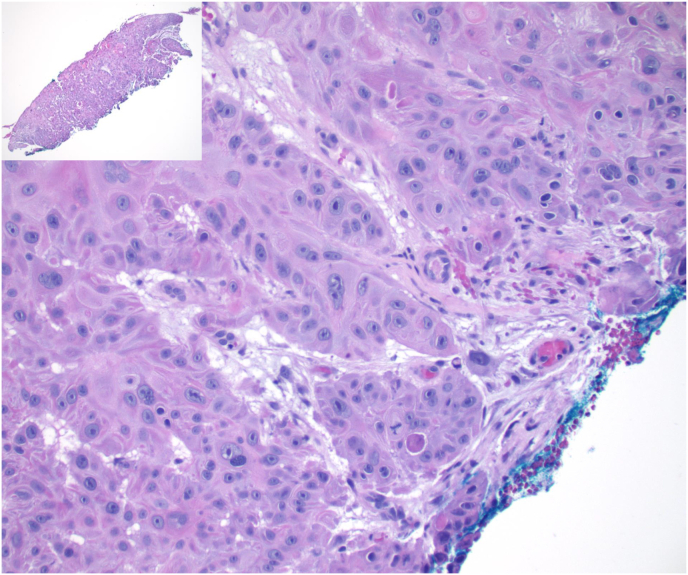
Microphotograph from the first biopsy shows well-differentiated squamous cell carcinoma with prominent nuclei and numerous mitotic figures. The tumor extends to the green inked margin of resection (Hematoxylin-Eosin, x400). Inset, Squamous carcinoma involves the entire specimen and extends to the blue inked margin of resection. (For interpretation of the references to colour in this figure legend, the reader is referred to the Web version of this article.)

A 76-year-old man presented to our service for evaluation of recurrent conjunctival squamous cell carcinoma (SCC). Five months prior to his visit at our clinic, the patient underwent an excisional biopsy of a lesion located on the nasal bulbar conjunctiva with an outside physician. The procedure included the excision of the lesion with a bare sclera technique, without wide marginal excision or cryotherapy. Subsequent histological examination revealed p40-positive, P16-negative, well-differentiated invasive SCC with positive excisional margins (Fig. 1). The patient also developed a recurrent conjunctival mass at the same location 2 months postoperatively. He was treated with 4 cycles of topical 5-Fluorouracil (5-FU) QID on a 1-week-on/1-week-off regimen, but the conjunctival lesion continued to enlarge. His other significant past medical history included treated chronic lymphocytic leukemia (CLL) and marginal zone lymphoma (MZL) of the right inguinal/thigh region, for which he received intravenous immunoglobulin infusions.

**Fig. 2:**
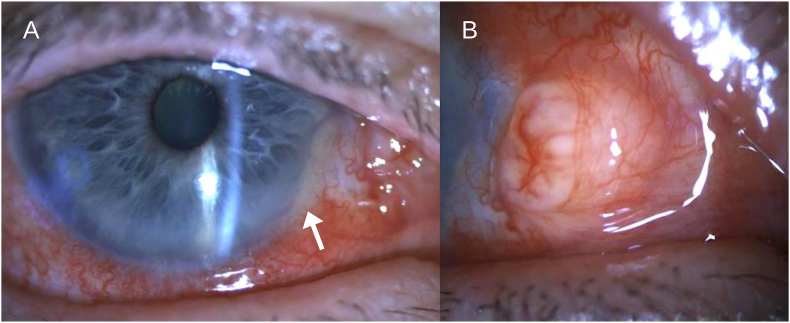
Slit lamp photograph at the first presentation at our unit, showing an elevated, amorphous, smooth-surfaced lesion on nasal conjunctival surface of the right eye. The lesion contained a few engorged vessels on the anterior margin and was found to be adherent to the underlying sclera.

**Fig. 3:**
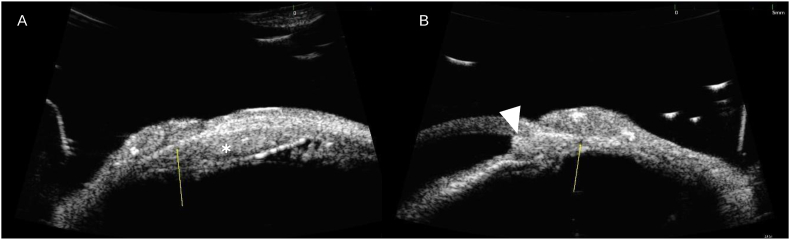
Ultrasound biomicroscopy in (A) Radial orientation, showing a hyperechoic conjunctival lesion inferonasally with underlying scleral thinning. The view to the ciliary processes inferiorly was obscured by the presence a biconvex hyperechoic lesion in the same region (asterisk). (B) Transverse orientation, showing blunting of the anterior chamber angle under the conjunctival lesion (arrowhead). In both images, a possible scleral connection between the ocular surface and intracameral lesions were identified (arrow).

On examination, his best corrected visual acuity (BCVA) was 20/25 in the affected right eye. There was 2+ conjunctival injection and an elevated, smooth amorphous lesion nasally measuring 4.7 mm and 3.9 mm in horizontal and vertical diameter, respectively (Fig. 2). The lesion contained engorged vessels on the anterior margin, and upon cotton tip applicator testing the inferior portion of the lesion was found to be adherent to the sclera. The cornea was clear and compact centrally, but there was mild dellen formation nasally adjacent to the conjunctival lesion. Additionally, the anterior chamber was notable for a non-shifting pseudohypopyon mixed with trace hemorrhage inferonasally corresponding to the conjunctival lesion. On gonioscopy, the pseudohypopyon was found to depress the iris surface without infiltration or vascularization. The intraocular pressure (IOP) measured with rebound tonometry was 14 mmHg. In contrast, the left eye was normal on examination, with BCVA of 20/20 and IOP of 10 mmHg. Pupils were equal, round, and reactive to light with no afferent pupillary defect bilaterally at this point. Ultrasound biomicroscopy (UBM) was performed over the right eye, which demonstrated a 2.23 mm-thick homogeneous hyperechoic conjunctival lesion involving the nasal corneal limbus (Fig. 3). In addition to scleral thinning locally, the anterior chamber was noted to have a biconvex lesion measuring 1.77 mm in thickness. The intracameral lesion showed a similar hyperechoic signal to the conjunctival lesion, and a possible connection between the two was also identified. These findings were suggestive of a recurrent conjunctival SCC with intracameral extension.

**Fig. 4:**
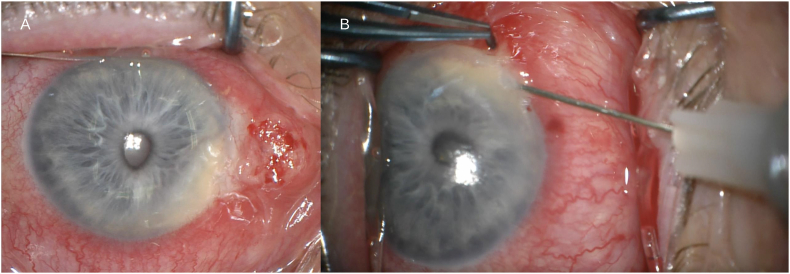
(A) Intraoperative photograph captured immediately after the incisional biopsy of the conjunctival lesion. Compared to the first presentation, the inferonasal intracameral extension has enlarged, and an inferior corectopia is observed. (B) Intracameral fine-needle aspiration with a 27G needle connected to a 1ml syringe.

**Fig. 5:**
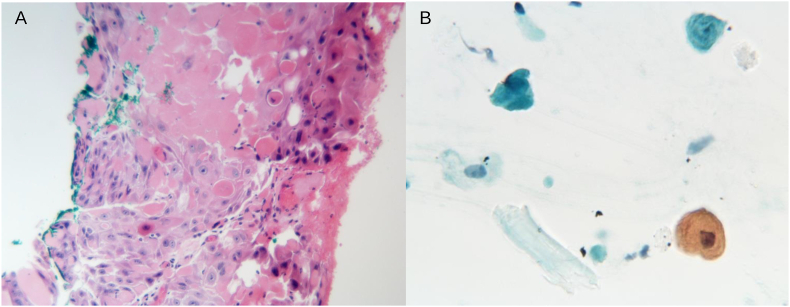
(A) Microphotograph of incision biopsy shows nests and strands of atypical squamous epithelium featuring dyskeratosis, enlarged pleomorphic nuclei, prominent nucleoli and mitotic figures. Inset, Squamous carcinoma involves the entire specimen and extends to the blue inked margin of resection. (B) Papanicolaou stain on intraocular aspirate shows atypical squamous epithelial cells with enlarged nuclei, prominent nuclei and abundant polygonal cytoplasm. There is a background of degenerative cells and anucleate squamous epithelium. There is dyskeratosis (orangophilic cytoplasm). There was no evidence of neutrophils (hypopyon) or lymphoma. Given the clinical picture, the findings are consistent with an intracameral extension of squamous cell carcinoma. (For interpretation of the references to colour in this figure legend, the reader is referred to the Web version of this article.)

Given the potential implication, an incisional biopsy of the conjunctival lesion consisting of three fragments, measuring total 2mm × 2mm, and intracameral FNA of pseudohypopyon were performed to confirm the suspicion of intraocular dissemination. Under peribulbar anesthesia (administered inferotemporally), The anterior portion of the conjunctival lesion adjacent to the limbus was debulked using Vannas scissors, and tissue was sent for histopathological analysis. Hemostasis was achieved with fine tip cautery. Additionally, a 27G needle connected to a 1ml syringe was used to enter the anterior chamber via the cornea at 4 o'clock (Fig. 4), and the pseudohypopyon was aspirated and sent for further evaluation. Subconjunctival vancomycin and dexamethasone were given at the end of the case. Histopathology of the conjunctival lesion showed large polygonal cells with individual cell keratinization, dyskeratosis, enlarged hyperchromatic nuclei, prominent nucleoli, and mitotic figures, confirming the presence of SCC which spanned the entire specimen (Fig. 5A). The intraocular aspirate contained scant atypical degenerative squamous epithelial cells (Fig. 5B), which in conjunction with the conjunctival histological findings was consistent with intracameral SCC dissemination. Notably, the anterior chamber aspirate did not contain any neutrophils or lymphocytes.

The patient was referred to the ocular oncology for further management. During this time, the conjunctival lesion continued to enlarge. The patient also developed an inferonasal corectopia that reducing the BCVA to 20/70, although the IOP was unaffected. Given the typically poor prognosis associated with intraocular SCC, enucleation was considered, but the patient expressed a strong preference for globe preservation. Consequently, a 12mm diameter Iodine-25 brachytherapy was recommended as a therapeutic trial.

**Fig. 6:**
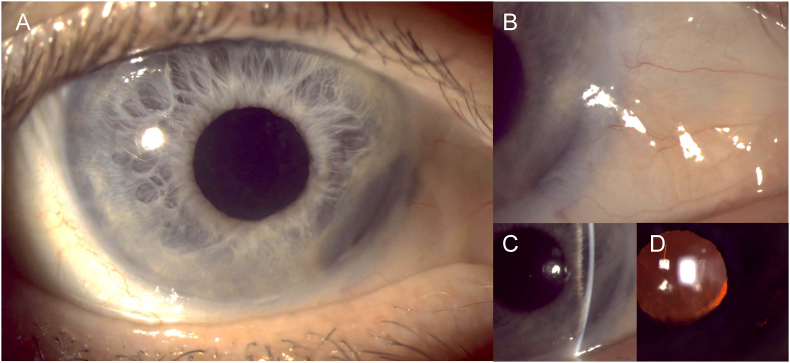
Clinical appearance at end of follow-up. (A) Low power slit-lamp photograph of the external ocular surface, showing mild degree of corectopia. (B) High power slit-lamp photograph of the nasal conjunctival area, showing the complete resolution of the malignant lesion. (C) Slit-beam examination illustrate the focal peripheral anterior synechiae corresponding to the location of the intraocular invasion. (D) Retro-illumination of the same area showing mild degree of transillumination defect after the resolution of the malignant lesion.

However, at an interim clinic visit four months after the incisional and liquid biopsies, the patient reported a reduction in lesion redness and size. Examination and repeat UBM showed reduced size of the external lesion and the pseudohypopyon. Brachytherapy was deferred, and the patient was further monitored. Over the course of the next 4 months, both the conjunctival lesion and the pseudohypopyon completely and spontaneously resolved. There were inferonasal iris stromal scarring and corectopia, as well as the formation of a nuclear sclerotic cataract which reduced the BCVA to 20/40. After a further 7 months of observation to ensure disease remission, and the patient underwent uncomplicated right cataract surgery in July 2023. One week after surgery, he achieved BCVA of 20/15, and has remained since until his most recent visit in March 2024, 27 months after incisional and liquid biopsies and 32 months since his first presentation (Fig. 6).

## 3 Discussion

We present a rare occurrence of spontaneous resolution of a recurrent and invasive conjunctival SCC after combined incisional and intracameral liquid biopsies. Conjunctival SCC is the most common non-pigmented malignancy of the ocular surface ^4^ and is part of the spectrum of ocular surface squamous neoplasia (OSSN). Risk factors for OSSN include solar UV exposure, smoking, vitamin A deficiency, chronic trauma or inflammation, allergic conjunctivitis, systemic or local immunosuppression, and infection with human immunodeficiency virus (HIV) or human papilloma virus. ^1^^,^^5^^,^^6^ Clinically, the presentation of OSSN varies greatly, and may appear as leukoplakic, gelatinous, or papillomatous lesions on the ocular surface. Lesions can be associated with feeder conjunctival vessels, along with conjunctival inflammation. ^6^ OSSN poses a significant clinical challenge, given its potential to result in vision loss, and in severe cases, even mortality. ^6^^,^^7^ For definitive diagnosis and as a curative treatment, an excisional biopsy with a “no touch” technique and large conjunctival margins (3–4mm) with cryotherapy is considered the standard of care. ^8^^,^^9^ Other less invasive diagnostic modalities include exfoliative cytology for superficial dysplastic lesions, in vivo confocal microscopy, or high-resolution optical coherence tomography (OCT). 10, 11, 12, 13 To evaluate the presence of intraocular tumor extension, ultrasound biomicroscopy (UBM) may be used due to its greater depth penetration. ^14^ Specific ultrasonographic findings suggestive of intraocular penetration include blunting of anterior chamber angle and thickening of uveal tissue.^13^, ^14^, ^15^

Traditionally, OSSN has been treated with wide surgical excision and intraoperative cryotherapy.^15^ However, disease recurrence has been reported to occur in as high as 33 % of cases with negative surgical margins, to 56 % with positive surgical margins. ^16^ Topical therapies, such as interferon-α2b, 5- fluorouracil, and mitomycin C are effective alternatives especially for the treatment of large, multifocal lesions as well as recurrent diseases. ^5^ Topical chemotherapy and immunotherapy can also be effective for the resolution of primary OSSN, with a success rate of 82 % for primary therapy with 5-fluorouracil. ^17^ Nevertheless, in our patient the recurrent conjunctival tumor was refractory to topical 5-Fluorouracil treatment, necessitating his referral to our center.

Intraocular extension of OSSN is a rare phenomenon. In a case series by Kaliki et al., only 2 % of participants suffered intraocular tumor extension, but 70 % of these cases were associated with history of previous OSSN excision and tumor recurrence which were seen in our case. ^15^ Other risk factors of intraocular extension include immunosuppression, limbal involvement, and nodulo-ulcerative conjunctival lesion. ^15^^,^^18^ Our patient's history of CLL/MZL, his systemic treatment of IVIGs, and the clinical appearance of his conjunctival lesion all aligned with these risk factors. Some studies have reported intraocular invasion of OSSN following intraocular procedures, such as filtering glaucoma surgery, clear corneal cataract surgery, or penetrating keratoplasty, suggestive of iatrogenic introduction of neoplastic cells in these cases. ^15^^,^^19^, ^20^, ^21^ However, prior to lesion resolution, our patient had not undergone any intraocular procedures.

Given the rarity of intraocular extension of OSSN, we proceeded with incisional biopsy of the conjunctival lesion to confirm the diagnosis of SCC tumor recurrence, as well as intracameral FNA of the pseudohypopyon to confirm the origin of the cellular deposit. Intraocular FNA was first introduced by Jakobiec et al., ^22^ and the technique is commonly used for the diagnosis of tumors of the uveal tract, including iris lesions in the anterior segment. ^23^ Several reports have described a variety of techniques of intracameral biopsy in the diagnosis of intraocular extension of OSSN, including 25-G vitrector-assisted iridectomy and anterior chamber paracentesis.^24^, ^25^, ^26^ However, to be best of our knowledge, the use of intracameral FNA in the diagnosis of intraocular extension of OSSN has not been previously described. We introduced the biopsy needle through a clear cornea approach, and we were careful to avoid any back-and-forth movement of the needle tip during the aspiration phase to reduce the likelihood of intraocular trauma, which may give rise to complications including traumatic cataract, corneal Descemet tears, and iris and/or angle hemorrhage. Indeed, any disruption of the blood-aqueous barrier in this case may result in the hematogenous spread of malignant cells which could have severe consequences.

After the incisional and liquid biopsies, our patient unexpectedly experienced spontaneous regression of the conjunctival tumor. Everson and Cole defined spontaneous regression as partial or complete resolution of a malignant lesion in the setting of inadequate or no treatment. ^27^ Spontaneous regression of tumors is infrequently encountered. Amongst 449 OSSN cases, Vempuluru et al. reported a 2 % spontaneous regression rate of OSSN lesions. ^28^ The regressing tumors predominantly arose in the nasal interpalpebral region and showed nodular or placoid morphology. The median tumor diameter was 4 mm, and the median time to resolution was 4 months after onset, although cases up to 17 months have also been observed. ^28^ Remarkably, these regressing tumors typically do not relapse up to 37 months of follow-up. ^28^^,^^29^ There are several hypotheses behind the mechanism of spontaneous tumor regression. In the series by Everson and Cole, 40 % of cases occurred following tissue biopsy, suggesting that immune response induced by local trauma could be a driver in tumor resolution. ^27^ Indeed, in a case report by Theotoka et al., ^29^ the authors reported complete OSSN tumor resolution after an incisional biopsy. They also proposed the “danger model,” wherein biopsy-associated trauma releases tumor-specific antigens, which are presented to naïve T-cells by local antigen presenting cells. Trauma to the tumor bed therefore signals danger to the host and initiates an immune-mediated attack on residual tumor cells. ^29^ Another suggested mechanism involves a reduction in tumor size post-biopsy, leading to a decrease in immune suppressive factors released by the tumor itself. ^26^ Other proposed mechanisms proposed include apoptosis driven by intrinsic or extrinsic pathways, inhibition by cytokines or growth factors, or inhibition of angiogenesis. Endocrine shifts due to hormonal therapy, pregnancy, or endocrine ablation have also been hypothesized to alter tumor cell survival. Heightened immunogenicity in the setting of infections, elimination of carcinogens, radiation exposure, and psychoimmunological mechanisms by “faith healing” or placebo drugs have also been discussed. 28, 29, 30, 31 In our case, we believe the conjunctival incisional biopsy was the primary driver of spontaneous resolution. IVIG therapy may have further altered the tumor microenvironment and enhanced immune surveillance. While intracameral FNA should further facilitate the process of disease resolution through reduction of disease burden, further studies are needed to validate this hypothesis.

Currently, there are no guidelines for vision restoration and treatment strategies following spontaneous resolution of OSSN. Indeed, after conducting a literature review on August 23rd, 2024, utilizing PubMed, Google Scholar, and Embase using the key words “procedure,” “ocular surface squamous neoplasia,” “spontaneous,” “resolution”, and other relevant synonyms, we did not find any prior reports of intraocular procedures after spontaneous regression of OSSN. Given the potentially devastating outcome of intraocular SCC, and that additional intraocular procedures may potentiate reactivation of intraocular OSSN if there were residual neoplastic cells, we intentionally monitored the patient for an extended period with a tapering follow-up scheme, at 1 month, 2 months, 3 months, and 6 months, to ensure disease remission before visual rehabilitation through cataract surgery. At each point, a careful anterior chamber and dilated fundus examination was performed, along with slit-lamp photography documentation and anterior segment OCT imaging, with additional UBM imaging at month 2, month 6 and month 18 after the incisional biopsy. As the risk of recurrence was uncertain, the patient was carefully counselled and was heavily involved in the decision-making process. Intraoperatively, care was taken to minimize any disruption of the ocular tissue in the area involved, including the avoidance of conjunctival dissection (in case of subtenon injections) and any iris manipulation, which explains our reluctance in performing any iridoplasty procedures. In the presence of a corectopic pupil, a monofocal intraocular lens was chosen instead of any refractive or diffractive premium lenses. Conventional post-operative care was given, with attention for any unusual amount of ocular surface and intraocular inflammation that may be suggestive of disease recurrence. Indeed, the patient remains under regular surveillance at this time.

In summary, spontaneous resolution of OSSN may occur after invasive investigational procedures, though the underlying mechanisms are not well understood. Cautious long-term monitoring of these patients is needed.

## CRediT authorship contribution statement

**Tanya Boghosian:** Writing – review & editing, Writing – original draft. **Promporn Patarajierapun:** Writing – review & editing, Writing – original draft. **Ben J. Glasgow:** Writing – original draft, Formal analysis, Conceptualization. **Carla B. Berkowitz:** Writing – review & editing, Writing – original draft. **Clemence Bonnet:** Data curation, Writing – review & editing. **Tara A. McCannel:** Resources, Data curation. **Brad D. Barrows:** Resources, Data curation. **Simon S.M. Fung:** Writing – review & editing, Writing – original draft, Supervision, Project administration, Conceptualization.

## 4 Patient consent

Consent to publish the case report was obtained. This report does not contain any personal information that could result in patient identification.

## Declaration of competing interest

The authors declare the following financial interests/personal relationships which may be considered as potential competing interests: TB, PP, BJG, CB, TAM, and BDD: none; SF is a consultant for Dompè US.; SXD is a consultant for Amgen, Cellusion, Kowa Research Institute, BrightMEM, Kala pharmaceuticals, and Claris Biotherapeutics, Inc.
